# Georeferencing of Personal Exposure to Radiofrequency Electromagnetic Fields from Wi-Fi in a University Area

**DOI:** 10.3390/ijerph17061898

**Published:** 2020-03-14

**Authors:** Raquel Ramirez-Vazquez, Sameer Arabasi, Hussein Al-Taani, Suhad Sbeih, Jesus Gonzalez-Rubio, Isabel Escobar, Enrique Arribas

**Affiliations:** 1 Applied Physics Department, Faculty of Computer Science, Engineering, University of Castilla-La Mancha, Avda. de España s/n, Campus Universitario, 02071 Albacete, Spain; raquel.ramirez@uclm.es (R.R.-V.); isabelmaria.escobar@uclm.es (I.E.); 2 School of Basic Sciences and Humanities, German Jordanian University, Amman Madaba Street, P.O. Box 35247, Amman 11180, Jordan; sameer.arabasi@gju.edu.jo (S.A.); hussein.taani@gju.edu.jo (H.A.-T.); suhad.sbeih@gju.edu.jo (S.S.); 3 Medical Science Department, School of Medicine, University of Castilla-La Mancha, C/ Almansa 14, 02071 Albacete, Spain; jesus.gonzalez@uclm.es

**Keywords:** electromagnetic fields, microenvironment, public exposure, radiofrequency and personal exposimeter

## Abstract

In the last two decades, due to the development of the information society, the massive increase in the use of information technologies, including the connection and communication of multiple electronic devices, highlighting Wi-Fi networks, as well as the emerging technological advances of 4G and 5G (new-generation mobile phones that will use 5G), have caused a significant increase in the personal exposure to Radiofrequency Electromagnetic Fields (RF-EMF), and as a consequence, increasing discussions about the possible adverse health effects. The main objective of this study was to measure the personal exposure to radiofrequency electromagnetic fields from the Wi-Fi in the university area of German Jordanian University (GJU) and prepare georeferenced maps of the registered intensity levels and to compare them with the basic international restrictions. Spot measurements were made outside the university area at German Jordanian University. Measurements were made in the whole university area and around two buildings. Two Satimo EME SPY 140 (Brest, France) personal exposimeters were used, and the measurements were performed in the morning and afternoon, and on weekends and weekdays. The total average personal exposure to RF-EMF from the Wi-Fi band registered in the three study areas and in the four days measured was 28.82 μW/m^2^. The average total exposure from the Wi-Fi band registered in the ten measured points of the university area of GJU was 22.97 μW/m^2^, the one registered in the eight measured points of building H was 34.48 μW/m^2^, and the one registered in the eight points of building C was 29.00 μW/m^2^. The maximum average values registered in the campus of GJU are below the guidelines allowed by International Commission on Non-ionizing Radiation Protection (ICNIRP). The measurement protocol used in this work has been applied in measurements already carried out in Spain and Mexico, and it is applicable in university areas of other countries.

## 1. Introduction

In the last two decades, due to the development of the information society, the use of information technologies has increased, including the connection and communication of multiple electronic devices such as mobile phones, computers, televisions, monitors, printers, etc., highlighting wireless fidelity (Wi-Fi) networks.

In addition, emerging technological advances such as the fourth and fifth generation networks, 4G and 5G, currently being deployed (this affirmation made refers to new-generation mobile phones that will use 5G), have led to a significant increase in the levels of personal exposure fields to electromagnetic radiofrequency (RF-EMF), causing an increase in the number of discussions about the possible adverse effects that these RF-EMF could have on human health [1,2,3], becoming the focus of our society.

In this context, the development of personal exposimeters opened a range of possibilities to be able to investigate in this field. Exposimeters are personal monitoring devices capable of measuring and registering orderly RF-EMF intensity measurements from different frequency bands [4,5] and providing a detailed description of the exposure [6] to be able to study and compare them with the guidelines allowed by International Commission on Non-Ionizing Radiation Protection (ICNIRP) [7].

Several studies have been developed for the evaluation of personal exposure to RF-EMF using different methodologies. Some of these studies are: point measurements [3,4,8,9], indoor and outdoor measurements during the development of daily activities [10,11,12], comparison of exposure levels between different zones and periods of the day [13,14], comparison of the levels of exposure recorded with the maximum levels permitted by international regulations [15], description of the levels of exposure and contribution of different RF-EMF sources [3,6,16,17,18], and monitoring of personal exposure to RF-EMF in microenvironments with the participation of volunteers [19,20,21,22] and with measurements made by the researcher himself [23,24,25,26].

In addition to the aforementioned studies, the increasing use of wireless Wi-Fi networks has prompted researchers to develop personal exposure studies to RF-EMF from this frequency band [27]. Among them, there is a summary on the state of the investigation of the possible effects of RF-EMF from the Wi-Fi network on public health [28], a review that shows seven effects of Wi-Fi in animals and human cells [29,30], and the results of a study conducted in primary and secondary schools [31]. The conclusions indicate that the registered values are below the exposure guidelines allowed by current international regulations [7]. The international reference level for the intensity of the wave with frequencies between 2 and 300 GHz is 50 W/m^2^ for occupational exposure and 10 W/m^2^ for exposure of the general public [7,32]. These maximum intensity values correspond to an electric field value between 137.3 V/m and 61.4 V/m, respectively, using rms values.

These studies are developed with personal exposimeters, lightweight and portable devices that among its main advantages are the easy handling for the participants in the study and the large amount of personal exposure data that can be obtained [16]. In addition to its advantages, we find some technical difficulties (effects on the human body, field strength and polarization rapidly varying over time-fading, calibrating equipment, etc.), methodological (measuring protocol), and of a data analysis-type (non-detects, using means, medians, etc.) [33,34,35]. These difficulties must be taken into account because they can affect the results of the research [16,36,37,38,39,40]. The most commonly used personal exposimeters are the EME SPY models 90 to 200 by Satimo, Brest France, SM 140 by Maschek (Bad Wörishofen, Germany), ExpoM and EME SPY 200 by Fields at Work (http://www.fieldsatwork.ch), capable of measuring up to 20 frequency bands (from 88 MHz to 5.85 GHz) and recording measurements in periods of between 2 and 255 s. Currently, thanks to technological advances we can find personal exposimeters that allow obtaining measurements in real time through the use of mobile applications such as Android, these meters are the EME SPY 200 manufactured by Satimo [41], EME Spy Evolution manufactured by Microwave Vision Group [42] and ExpoM-RF manufactured by Fields at Work GmbH derived from ETH Zurich (Microwave Vision Group, Zürich, Switzerland).

The main objectives of the studies with personal exposimeters are, first of all, to know in detail the personal exposure of the population, second, to measure exposure levels in different microenvironments [15,16,20,24,43,44,45,46], and third, to make sporadic and point measurements, and to design models to estimate exposure levels [12,47,48,49,50,51,52].

Despite the results obtained in these studies, a large part of the population remains concerned as diseases of unknown etiology appear [53,54,55,56], which is why it is necessary to continue researching to try to respond to these concerns.

When Pall’s work was published [30], whose title is “Wi-Fi is an Important Threat to Human Health” it was interesting and worrisome, and therefore it motivated us to write a comment [29]. We really wanted to check if people using a Wi-Fi network are under threat to their health. Dr. Pall reviewed seven possible adverse effects of 2.4 GHz radiofrequency (Wi-Fi). Each of them was documented quite comprehensively. Those seven effects are: cellular DNA damage, changes in testis structure, lowered sperm count/quality, neurological/neuropsychiatric effects, apoptosis/cell death, calcium overload, endocrine effects, oxidative stress, and free radical damage.

**Figure 1:**
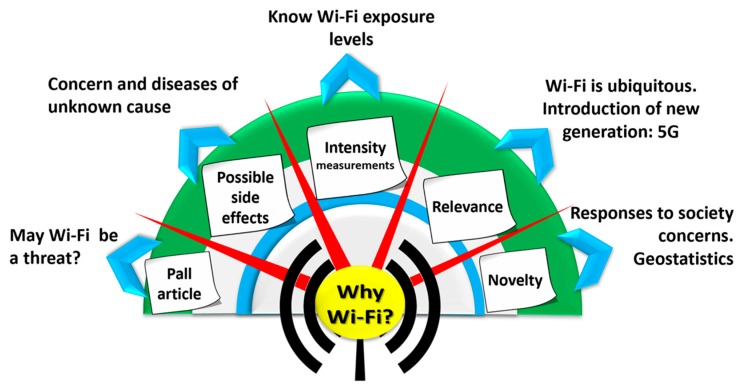
Reasons that motivated us to measure levels of exposure to highlighting wireless fidelity (Wi-Fi) electromagnetic radiofrequency (RF-EMF).

Derived from the above, one of our approaches was to consider how could one verify what Dr. Pall describes in his article? Why perform a study on the Wi-Fi network? Graphically, one can see in the Figure 1 our reasons. Could we contribute something to clarify this uncertainty? With the resources we have, we intend to measure the intensity levels of radiofrequency electromagnetic fields to which we are exposed due to the Wi-Fi wireless network, which we use regularly. This unknown motivated and gave rise to this work about the measurement of personal exposure to radiofrequency electromagnetic fields from Wi-Fi (which is ubiquitously permeating all our space, in which we are submerged, and navigating in a sea of radiofrequency waves that surrounds us completely) in order to verify compliance with the maximum permitted by international regulations [7].

For this reason, we focused on this work developed in Jordan, because most of the studies of personal exposure to RF-EMF have been developed in Europe, and some are beginning to be developed in other continents, especially this one that is developed in the Asian continent. Due to the interest in this type of signal and the lack of knowledge of the levels of exposure to RF-EMF from Wi-Fi in the university area of German Jordanian University, we have carried out this study considering the measurement protocol proposed and used in other studies [6,16,20].

Performing measurements of all locations in a study area to determine concentrations or the magnitude of a phenomenon in order to know personal exposure levels to RF-EMF is difficult, but it is likely to measure in locations of certain samples, strategically scattered in such a way that values can be assigned to the rest of non-measured locations based on the measurements already performed. For this purpose, there are geostatistical analysis methods such as Kriging interpolation, permitting to interpolate values in the sampling areas where no measurements are performed, bearing in mind the structure of spatial correlation. In this work, this spatial statistical technique has been applied for the analysis and mapping of RF-EMF intensity levels registered in the study area.

The main objective of this research was to measure the personal exposure to radiofrequency electromagnetic fields coming from the Wi-Fi wireless connections in the frequency ranges of 2400–2500 MHz and 5150–5850 MHz, in the university area of German Jordanian University (GJU) and to make georeferenced maps of the recorded intensity levels and compare them with the basic international restrictions.

## 2. Methods

### 2.1. Study Area

The measurements were performed in the campus at German Jordanian University (GJU), located in Amman, Jordan. Jordan is an Asian country located in the Middle East region, its capital and most populous city is Amman, a mountainous region of north-western Jordan, with geographical coordinates of Latitude: 31° 57′ 0″ N and Longitude: 35° 56′ 0″ E. GJU is located at the geographical coordinates of Latitude: 31° 46’ 36” N and Longitude: 35° 48’ 9” E.

**Figure 2:**
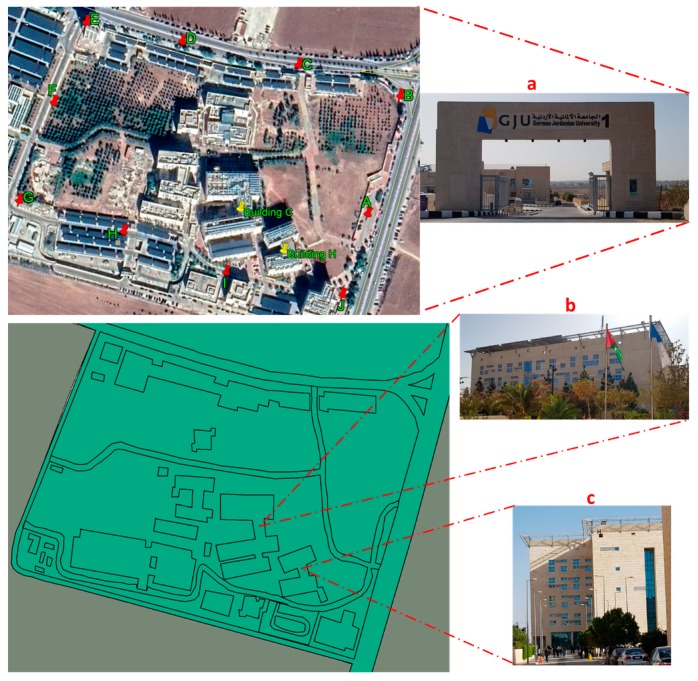
Map of the university area of GJU and measured buildings. (**a**) GJU, (**b**) building C and (**c**) building H.

To perform the measurements, the study area was divided into three microenvironments: (a) around university area of GJU; (b) around the building C, classrooms and laboratories where students are concentrated; and (c) around the building H, professors offices area (Figure 2).

**Figure 3:**
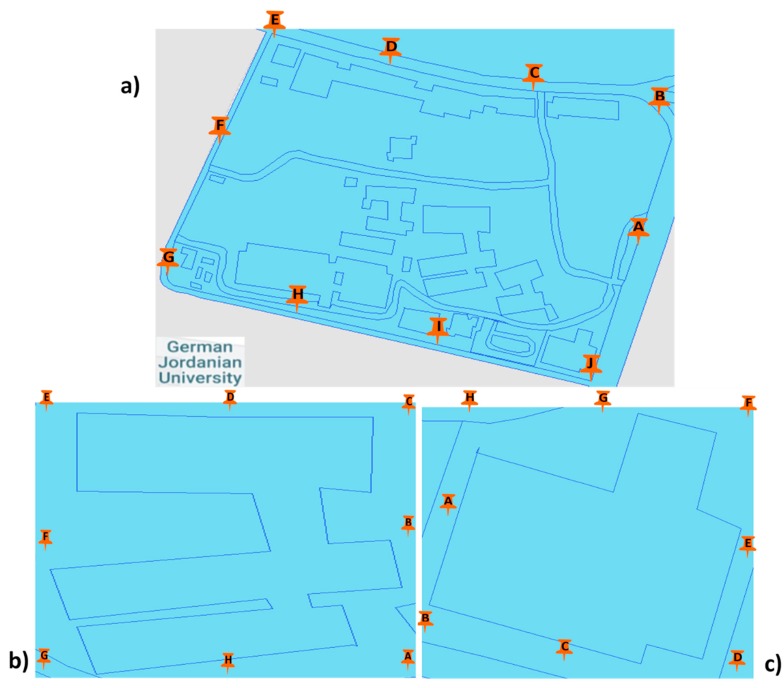
Location of the measured points in the study area: (**a**) 10 points (A-J) around GJU, (**b**) 8 points (A-H) around building C and (**c**) 8 points (A-H) around building H.

Spot measurements were made outside in the campus at German Jordanian University, where ten strategic points were chosen. Eight strategic measurement points were also selected around buildings C and H. In each of the two buildings, eight strategic measurement points were selected, see Figure 3.

### 2.2. Exposimeter Measurements

Two Satimo EME SPY 140 (Brest, France) personal exposimeters were used, duly calibrated by TEMSYSTEM (Madrid, Spain, www.temsystem.es), and configured in the same way before carrying out the measurement process to ensure the accuracy of the measurements in relation to time. The EME SPY 140 measures 14 frequency bands between 88 MHz and 5 GHz and records up to 12,540 measurements during periods lasting between 4 and 255 s. The minimum value detected by the exposimeter in each frequency band is, in FM (radio broadcast transmitter): 6.631 µW/m^2^; TETRA (mobile communication for closed groups) and TV4&5 (broadcast transmitter): 0.265 µW/m^2^; GSM (global system for mobile communications), DCS (digital communications system), DECT (digital enhanced cordless telecommunications), UMTS (universal mobile telecommunications system), Wi-Fi 2G (wireless local area network): 0.066 µW/m^2^; and in TV3 (broadcast transmitter), WiMAX (worldwide interoperability for microwave access), Wi-Fi 5G (wireless local area network): 1.06 µW/m^2^.

As indicated, the objective of this study was to measure personal exposure to RF-EMF from the Wi-Fi frequency band, however, we have also included the total average exposure value from all bands measured by the exposimeter. Despite having the data of the 14 bands measured by the exposimeter, we have focused our study on the data of the Wi-Fi bands in the frequency ranges of 2400–2500 MHz and 5150–5850 MHz. We have included the three radiofrequency bands that contribute the most, in addition to Wi-Fi, and the total of the 14 bands measured by the exposimeter, the total result of all periods.

The exposimeters were configured to measure every 4 s for a period of three min, but only 2 min measurements were considered (to avoid errors or interferences, 30 initial s and 30 final s were eliminated) of each of the 26 points, 10 points outside the university area, 8 points outside building H, and another 8 outside building C. This means that, at each point, 30 measurements were obtained from each of the two exposimeters, of which the average was subsequently calculated.

For the measurement protocol it was considered to set up the personal exposimeter in order to register measurements every 4 s, since one of the features of the device is the registration up to 12,450 measurements during 4-s and 255-s periods; in this case, the lower limit has been taken into account with the aim of obtaining the higher amount of measurements in a continuous way during the measurement period, that is to say, 15 registers per minute. It was decided to measure in a 3-min period for every point (sample) in order to eliminate 30 s at the end of the measurement period in every point, solely considering measurements registered in the central 2 min as valid, so as to avoid any interference at the beginning and the end of the procedure. Besides, to minimize laterality problems, the exposimeter was elevated over the head to avoid the effect of the body on the measurement. It was considered that exposure would not change at a height of 1 or 2 m.

Both devices employed were previously calibrated by the company TEMSYSTEM (Madrid, Spain, www.temsystem.es), and both calibration and verification of devices are performed every two years.

**Figure 4:**
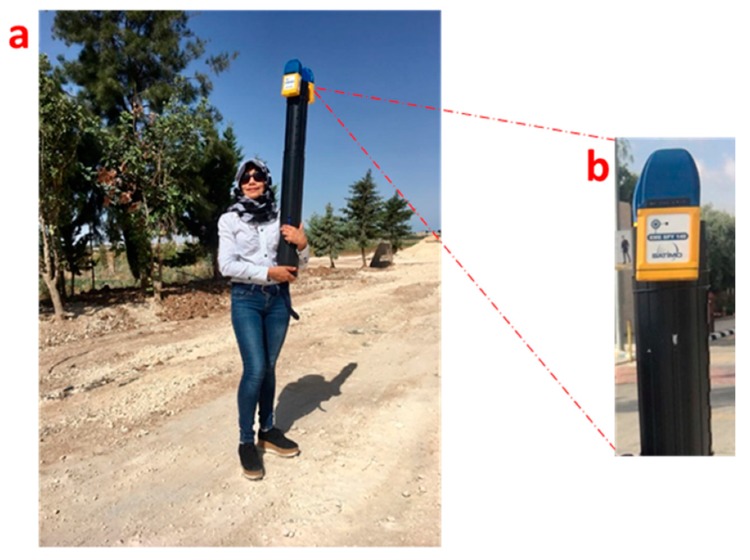
(**a**) Measurement method using two exposimeters placed above the head of the measurement researcher and (**b**) personal exposimeter.

The two exposimeters were placed in a cardboard tube (one on each side) so that, during the measurement process, the exposimeter was taller than the head of the researcher, thus avoiding the effect of the body [33,34,56] (Figure 4).

### 2.3. Measurements Protocol

A member of the research team toured the study area with the two exposimeters placed in the cardboard tube accompanied by a GJU professor who carried a Global Positioning System (GPS) to record the coordinates of the points where the measurements were made, as well as a plastic wrist watch and a personal diary where the start and end time, the measured point, and respective coordinates were recorded.

Both the GPS and the watch were made of plastic, in order to avoid contact or some interference with metals. Further, during the measurement process, no mobile phone was used. All electronic devices (mobile phone, smartwatches) were completely disconnected, except for the GPS an assistant was carrying.

The measurements were carried out in two periods of the day, in the morning from 8:00 a.m. to 10:00 a.m. and in the afternoon from 1:00 p.m. to 3:00 p.m., at which time during the week there is more concentration of people in the study area. The measurement process was for four different days, two days on the weekend and two days on weekdays. A total of 12,480 data were obtained for each exposimeter, including days and areas measured.

### 2.4. Analysis of the Measurements and Exposure Maps

We have chosen to study personal exposure to RF-EMF through wave intensity, which is expressed in W/m^2^. Some studies have used different submultiples of this measure, such as µW/cm^2^, mW/m^2^, although other studies have also used electric fields in V/m. Because the typical values and the equipment are extremely sensitive, in this study the µW/m^2^ have been used as a unit of measurement, values that are easy to process and represent, because they vary mainly between 0 and 1000.

At the conclusion of the measurement process, the cleaning and classification of the measurements by period, area and measured point was performed. The statistical analysis of the data was performed with the EME Spy Analysis Software version 3.20 of EME SPY personal exposimeter by Satimo (Brest, France), IBM SPSS Statistic version 22 software by IBM Corporation and ArcGIS version 10.6 software by ESRI Spain.

The average levels of personal exposure of the Wi-Fi frequency band (2400–2500 MHz and 5150–5850 MHz frequency ranges) of each measured point and totals were calculated, as well as the average levels from the total of the bands measured by the exposimeter. The measurements were classified by point and area measured: 10 points around of GJU (A–J), 8 points around of building C (A–H) and 8 points around of building H (A–H).

A 60% of nondetect data was found throughout the band subject to measurement. For the management of this values acquired when no signal is detected (nondetects), we assume that the actual field in that moment should approach to zero, therefore management of non-detected values was done replacing non-detected values by the exposimeter detection limit divided by 2 (method 2) and the corresponding citation was included [40].

With the use of Google Maps by Microsoft and Google Earth Pro by Google LLC, the georeferencing of the area and measured points was performed. Subsequently, with the use of geographical coordinates and average exposure values recorded in μW/m^2^, georeferenced intensity maps were prepared, interpolating the values with the help of ArcGIS software.

As stated in the introduction, with the aid of interpolation methods, in this work, the intention has been to provide maps of RM-EMF exposure intensity levels registered in the points subject to measurement and not measured before. For this purpose, sampling points coordinates were registered, together with intensity levels measured, and subsequently stored in a geographic information system (GIS). Eventually, with the help of ArcGIS software, levels of the measured sample and non-measured points were interpolated, using spherical Kriging.

## 3. Results

### 3.1. Measurements

Considering the three areas of study during a four-day period, both in the morning and afternoon, the average total RF-EMF intensity from the Wi-Fi band was 28.82 μW/m^2^ and that from the total of the 14 bands it was 1598.8 μW/m^2^ (Table 1).

In addition to the total intensity to RF-EMF from Wi-Fi recorded during the entire measurement period and in the different microenvironments, we have compared this value with the total of each of the bands measured with the exposimeters, and in Table 2 we can see the three bands that contributed the most, in addition to Wi-Fi.

The average intensity from the Wi-Fi band registered in the 10 points at the university area of GJU was 22.97 μW/m^2^, the one recorded in the eight points of the H building was 34.48 μW/m^2^ and the one recorded in the eight points of building C was 29.00 μW/m^2^.

The measurements were made for two weeks, last week of June and first week of July 2018. Each exposimeter measured a total of 416 min and a total of 12,480 measurements were obtained and averaged. Measurements from the 14 frequency bands were recorded, but in this article, we only present those recorded by the Wi-Fi band.

### 3.2. Temporal Measurement of Personal Exposure to RF-EMF

**Figure 5:**
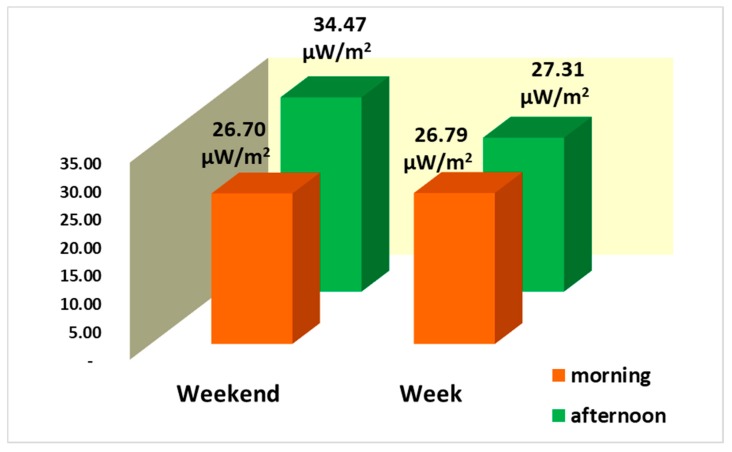
Total average intensity to RF-EMF from the Wi-Fi band, on the week and weekend (μW/m^2^).

Figure 5 shows the total average of the measurements recorded on weekends and weekdays for each period measured, in the morning and in the afternoon, from the Wi-Fi band. If we compare the means recorded by measured day and time of the day, we observe that during the week the highest value was in the afternoon, but there was not great difference compared to the morning values; and on the weekend, the highest value was in the afternoon. This is because the highest concentration of people who are connected to the Wi-Fi network, in both periods, is in the afternoon.

### 3.3. Spatial Measurement of Personal Exposure to RF-EMF

**Figure 6:**
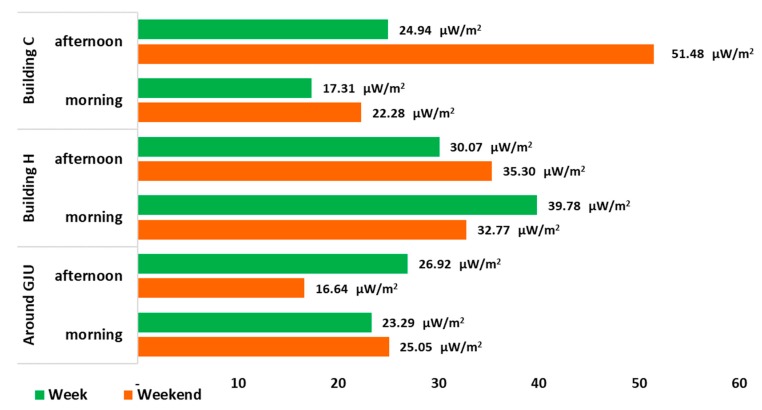
Wi-Fi exposure of RF-EMF from the mean contributions per measured area, indicating morning and afternoon (intensity in μW/m^2^).

When comparing the average of the RF-EMF intensity of the means registered by measured area, type of day and time of the day, from Wi-Fi, we observe that the maximum value was recorded in building C, in a weekend in the afternoon, with a value of 51.48 μW/m^2^; and the minimum value was recorded in the measurements made around at the university area, in a weekend in the afternoon, with a value of 16.64 μW/m^2^ (Figure 6). Building C is an area where classrooms and laboratories are located, therefore, more Wi-Fi antennas are installed, and on weekends in the afternoon, students and professors frequent this area to connect their computers or mobile phones to perform different academic or personal tasks.

### 3.4. Spatial Measurement of Personal Exposure to RF-EMF: Week and Weekend, and Morning and Afternoon.

In Table 3, Table 4 and Table 5, the RF-EMF intensity levels from the Wi-Fi antennas, recorded in each of the measured microenvironments, by day and time of the day measured, are shown in each of the University area buildings.

**Figure 7:**
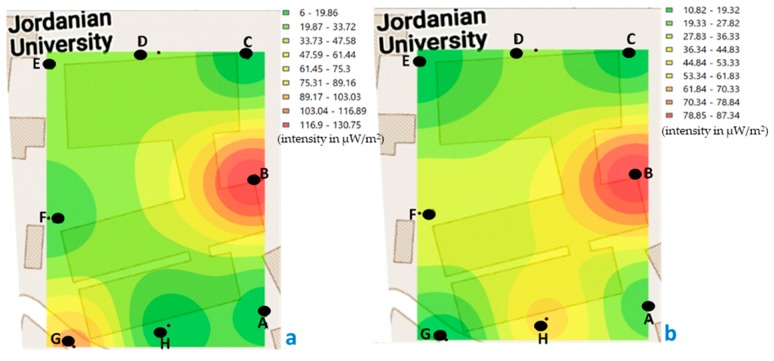
Georeferenced map with the levels of exposure recorded in building C (classrooms and laboratory, points A–H), in the morning. (**a**) weekend and (**b**) during the week (μW/m^2^).

**Figure 8:**
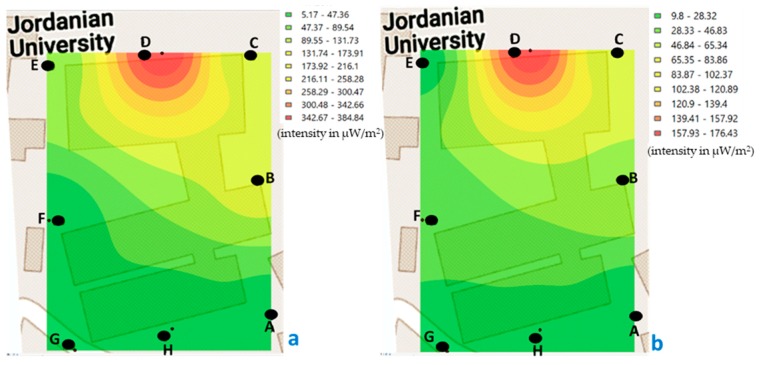
Georeferenced map with the levels of exposure registered in building C (classrooms and laboratory, points A to H), in the afternoon. (**a**) weekend and (**b**) during the week (μW/m^2^).

The minimum value recorded in building C, during morning measurements was 7.48 μW/m^2^ and the maximum of 130.75 μW/m^2^ (Figure 7); while, in the afternoon, the minimum was 5.17 μW/m^2^ and the maximum was 384.86 μW/m^2^ (Figure 8).

If we compare the values recorded at the measured points of building C, in the morning (Figure 7), we observe that the highest values are at point B, both for the weekend and during the week.

In Figure 7, the intensity levels in each of the points measured in building C are represented graphically (in the morning, on weekends and weeks), and from that set of measurements an analysis of interpolation to predicting unknown values at other locations and represent areas with low and high intensity from all area. If we focus on Figure 7a, we observe that between H and A points (in the lighter green zone) the exposure levels have a value between 19.87 and 33.72 μW/m^2^. With the objective to verify the validity of the kriging, we have measured that same day in the afternoon on the weekend, in the middle of the two points indicated (H and A), obtaining a value of 24.76 μW/m^2^. Therefore, we can verify that the interpolation method used calculates acceptable values. In the same way it was measured out in the center of the area that represents building C (the same day and time of day, Figure 7a), and it was obtained that the intensity level was 51.13 μW/m^2^. With the above data, we can verify that it is possible to interpolate using known values of points measured in a certain study area, to know the areas with low and high value, in this case, of personal exposure to RF-EMF.

If we look at Figure 7b, we can see that, as in Figure 7a, the highest value is at point B, and that the yellow color extends mostly in the center area of the image, showing values between 36.34 and 61.83 μW/m^2^. In the same way, it was measured in the middle point of this study area, obtaining a value of 37.09 μW/m^2^.

When comparing the values recorded in the measured points of building C, in the afternoon (Figure 8), we observe that the highest values are at point D, both for the weekend and during the week. In this building C, two signal boosters for mobile service providers are installed on top of the building.

In the areas where the highest values are concentrated, both in Figure 7 (point B) and Figure 8 (point D), there are laboratories, in which some Wi-Fi antennas are installed allowing greater connectivity of laboratory equipment, as well as, computers and smartphone of students and professors. In both measured periods, there were many people connected using the Wi-Fi network.

**Figure 9:**
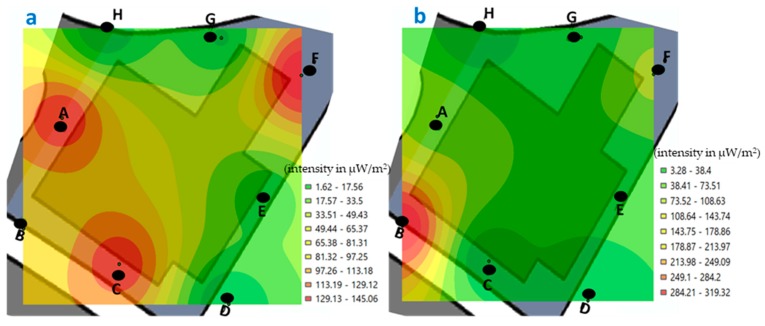
Georeferenced map with the levels of exposure registered in the H building (professors’ offices, points A–H), in the morning. (**a**) weekend and (**b**) during the week (μW/m^2^).

**Figure 10:**
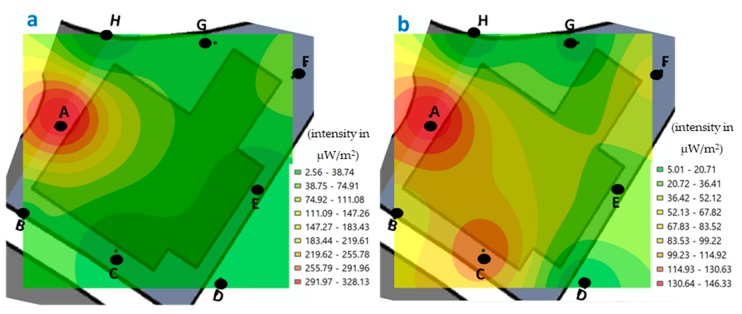
Georeferenced map with the levels of exposure registered in the H building (professors’ offices, points A–H), in the afternoon. (**a**) weekend and (**b**) during the week (μW/m^2^).

The minimum value recorded in building H, during the morning measurements was 1.62 μW/m^2^ and the maximum of 319.33 μW/m^2^ (Figure 9). Meanwhile, in the afternoon, the minimum was 2.56 μW/m^2^ and the maximum was 328.14 μW/m^2^ (Figure 10).

If we compare the values recorded at the measured points of building H, in the morning (Figure 9), we observe that the highest values for two periods, was at point B during the week (Figure 9b). However, the highest value during the week (Figure 9a), was at point A, C, and F, at which time professors were connected, because as indicated, in this building are the professors’ offices.

If we compare the values recorded at the measured points of building H, in the afternoon (Figure 10), we observe that the highest values are at point A, both for the weekend and during the week.

In building H, specifically, where the points A, B, and C are marked are close to the cafeteria area where there is a high number of students and hence an increase in the use of connectivity to the local Wi-Fi network of building H.

**Figure 11:**
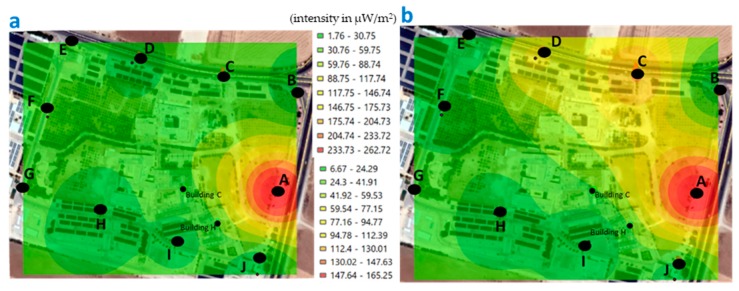
Georeferenced map with the levels of exposure recorded around the university area of the GJU, in the morning (points A-J). (**a**) weekend and (**b**) during the week (μW/m^2^).

**Figure 12:**
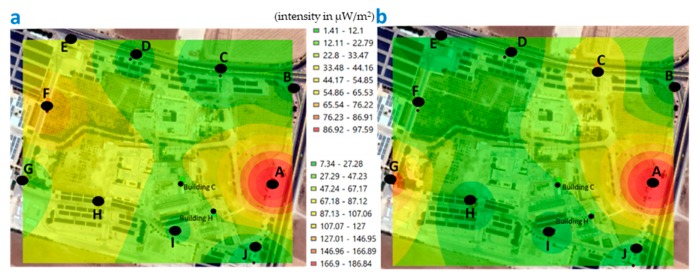
Map georeferenced with the levels of exposure recorded around the university area of GJU, in the afternoon (points A–J). (**a**) weekend and (**b**) during the week (μW/m^2^).

The minimum value recorded around the university area of GJU, during the morning measurements was 1.75 μW/m^2^ and the maximum of 262.74 µW/m^2^ (Figure 11); while, in the afternoon, the minimum was 1.41 μW/m^2^ and the maximum was 186.85 μW/m^2^ (Figure 12).

If we compare the values recorded at the points measured around the university area of GJU, in the morning (Figure 11), we observe that the highest values are at point A, both for the weekend and during the week.

When comparing the values recorded in the points measured around the university area of GJU, in the afternoon (Figure 12), we observe that the highest values are at point A, both for the weekend and during the week. This point A identified around the university area is closer to the main mobile towers outside GJU campus.

## 4. Discussion

This work was developed in the university area at GJU, personal exposure to Electromagnetic Fields from the Wi-Fi band was measured, to know if the intensity levels meet the guidelines allowed by international regulations. It is the first study of these characteristics that is carried out in this university. Two personal exposimeters Satimo EME SPY 140 (Brest, France) were used, calibrated by the company TEMSYSTEM (Madrid, Spain, www.temsystem.es). The measurements were similar, therefore, an average of the measurements by both exposimeters was obtained.

The total average of the RF-EMF personal exposure from the Wi-Fi band registered in the three study areas and during the four days measured was 28.82 μW/m^2^. The average total exposure recorded around the entire university area of GJU was 22.97 μW/m^2^, the one recorded in building H was 34.48 μW/m^2^ and the one recorded in building C was 29.00 μW/m^2^. The exposure levels from Wi-Fi are very changing, for example, in Table 3 we can see that in the morning it ranged between 7.48 and 130.75 μW/m^2^, whilst in the afternoon it was between 5.17 and 384.86 μW/m^2^. This is due to the greater or lesser use of GJU students’ and teachers’ wireless network. The same can be observed in Table 4 and Table 5.

Building C is an area where classrooms and laboratories are located, therefore, more Wi-Fi antennas are installed, and on weekends in the afternoon, students and professors frequent this area to connect their computers or mobile phones to perform different academic or personal tasks.

**Figure 13:**
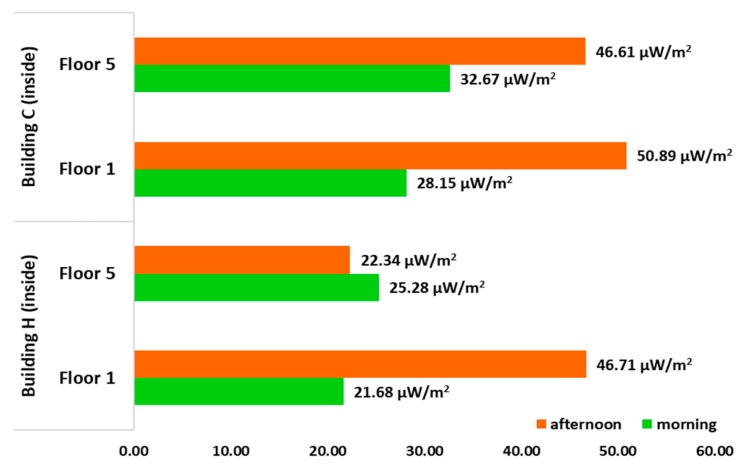
Wi-Fi exposure of RF-EMF from the mean contributions inside building H and C, indicating afternoon and morning (μW/m^2^).

In order to compare the total personal exposure to RF-EMF obtained around each of the buildings during the period measured, measurements were also made within the H and C building, on the first and fifth floors. The total recorded average is shown in Figure 13. As we can see, the highest values within the building are a little higher on the first floors both building H and C, during the afternoon which is when there is the highest concentration of students, and where there are mostly more people connected to mobile phones and computers; while, during the morning, the average recorded was smaller, because in that period of time there is a lower concentration of people connected inside the building.

If we compare our results obtained in the Wi-Fi band with the results of the studies reviewed by Jalilian [57] and some other studies that considered measurements from Wi-Fi frequency band (Table 6), we can observe that our results are higher, except the result for Belgium which was 38.33 μW/m^2^ [43].

Furthermore, if we compare these results with those presented by Ramirez-Vazquez [20] from measurements in a Spanish town (Albacete), where the minimum value from Wi-Fi was 22.8 μW/m^2^ and maximum value was 86.9 μW/m^2^, in this case, the results of our study are similar with minimum value. However, if we make a comparison with the results published by Khalid [31], who reported a maximum time-averaged intensity from a laptop of 220 μW/m^2^ at a distance of 0.5 m in a laboratory in Oxfordshire, we can observe that the values of our work are smaller.

In this study, we have considered the measurement protocol proposed and used in some other studies [6,16,20,47], and this measurement protocol we have applied in other countries, such as Spain, Mexico, and Brazil. We believe it can be useful and can be applied in other, similar studies in different countries. The research protocol that has been taken as a reference in the development of this type of studies has been the one proposed by Röösli [6], and the protocol similar to ours is the one that has been used by Aerts [47], which was used to accurately map the radiation exposure from global system for mobile communications technology at 900 MHz (GSM 900) using a personal exposimeter.

Due to the deployment of the new 5G generation, when it is implemented in Jordan, we want to repeat, in the near future, the measurements in this same area of study (around GJU, building C, and building H), and make a comparative analysis between both measurement periods.

Referring to previous studies by the authors of this work [2,20,23,24,25,29,35], in this research, some different aspects were studied and added, that is to say, measuring in certain points of a certain town area where no similar measurements had been performed, then focusing the study on Wi-Fi band. In addition, in the elaboration of intensity maps georeferenced with interpolated data, measurements were represented in order to identify which points were the highest ones and to search for a possible explanation. We consider that this aspect is new in our study area, and we want to keep investigating in other microenvironments, looking for an improvement in the protocol of measurement and data analysis methods.

## 5. Conclusions

In the Wi-Fi band, the highest average values recorded in the university area of GJU are well below the maximum levels allowed by International Commission on Non-Ionizing Radiation Protection (ICNIRP). Our measurements are 0.0010% of the maximum allowed on urban land, which is 10 W/m^2^.

The measurement protocol used in this work has been applied in measurements performed in two different countries, namely Spain and Mexico, and we believe that it may be useful to apply it in university areas of other countries. We intend to use it in other countries to perform the respective comparative analyses to verify compliance with the corresponding regulations.

As mentioned in the introduction, the levels of RF-EMF exposure are increased, due to the massive use of information technologies that increasingly demand a greater number of connected users. The international organizations such as ICNIRP have established basic restrictions and permitted reference levels for the RF waves intensity based on results obtained in studies already performed in this field, with the aim of providing a true reference for population, and adopting corrective measurements in the case of exceeding those guidelines. Although the studies carried out demonstrate compliance with these legal guidelines, it is necessary to continue researching and developing this type of studies in other countries, as there are still no concrete conclusions on this field. Therefore, researchers recommend conducting RF-EMF exposure assessments, not only in places where they have already developed, but also, and more interestingly, in places that have never been evaluated, such as the university area of GJU.

After measurements at German Jordanian University, as authors we are certain that Wi-Fi is not likely to be responsible for effects on human health commented on by Pall [30] who states and cites seven possible adverse effects of 2.4 GHz radiofrequency (Wi-Fi). Levels measured are in the region of the millionth part of maximum permitted value, that is to say, a −55-dB signal, meaning it is very low. If they were sound waves, the measurement would be inaudible to us when compared with the maximum permitted by ICNIRP.

## Figures and Tables

**Table 1:** Intensity from Wi-Fi band and the total exposure from the 14 bands.

Wi-Fi Bands	Total of 14 Bands
28.82 μW/m^2^	1598.8 μW/m^2^

**Table 2:** The three bands that contribute most to the total intensity of Radiofrequency Electromagnetic Fields (RF-EMF).

Bands	Frequency (MHz)	Intensity in μW/m^2^
DCS DL (Digital Communications System)	1805–1880	568.25
GSM DL (Global System for Mobile Communications)	925–960	556.52
UMTS DL (Universal Mobile TelecommunicationsSystem)	2110–2170	263.20

**Table 3:** Measures recorded around building C (classrooms and laboratory), values expressed in μW/m^2^.

Point	Morning		Afternoon	
	Weekend	Week	Weekend	Week
A	13.41	22.17	23.16	9.80
B	130.75	87.34	150.48	62.38
C	6.00	13.06	137.21	69.02
D	44.20	28.97	384.86	176.44
E	37.92	10.82	93.23	15.08
F	21.95	43.83	5.17	29.67
G	94.82	14.08	19.59	12.82
H	7.48	56.72	10.05	23.80

**Table 4:** Measures recorded around building H (professors’ offices, points A to H), values expressed in μW/m^2^.

Point	Morning		Afternoon	
	Weekend	Week	Weekend	Week
A	126.08	92.70	328.14	146.33
B	82.56	319.33	69.85	75.58
C	125.19	7.62	6.04	112.21
D	1.62	3.35	4.69	5.78
E	24.41	41.74	13.97	49.13
F	145.06	119.36	94.74	72.54
G	16.17	49.11	44.75	14.52
H	3.17	3.28	2.56	5.01

**Table 5:** Measures recorded around the university area German Jordanian University. (GJU, points A to J), values expressed in μW/m^2^.

Point	Morning		Afternoon	
	Weekend	Week	Weekend	Week
A	262.74	165.26	97.60	186.85
B	11.08	15.92	7.76	12.98
C	39.62	113.30	14.02	84.52
D	18.21	80.55	19.66	32.39
E	50.17	6.67	36.73	7.34
F	48.66	11.11	65.67	35.40
G	33.07	36.67	26.02	134.96
H	12.79	11.88	42.29	15.22
I	22.96	7.99	21.56	20.91
J	1.75	16.48	1.41	7.82

**Table 6:** Results of some reviewed studies that considered measurements of the Wi-Fi frequency band (μW/m^2^).

Author	Countries	Mean (μW/m^2^)
Birks et al., 2018 [1]	Denmark (all environments)	1.19
	Netherlands (all environments)	1.19
	Slovenia (all environments)	0.53
	Switzerland (all environments)	0.53
	Spain (all environments)	6.50
Roser et al., 2017 [58]	Switzerland (all environments)	1.19
Valic et al., 2015), 2015 [59]	Slovenia (all environments)	4.77
Röösli et al., 2010 [6]	France (all environments)	1.92
Massardier-Pilonchery et al., 2019 [60]	France (all environments)	0.15
Hardell et al., 2017 [61]	Sweden (School)	3.32
Roser et al., 2017 [58]	Switzerland (school)	1.19
Hedendahl et al., 2017 [62]	Swedish (outdoor, old town)	0.13
Gonzalez-Rubio et al., 2016 [24]	Spain (outdoor)	0.53
Aminzadeh et al., 2016 [43]	Belgium (office indoor, urban)	38.33
Our measurements	Jordan (university area)	28.82
